# Conventional maxillary denture versus maxillary implant‐supported overdenture opposing mandibular implant‐supported overdenture: Patient's satisfaction

**DOI:** 10.1002/cre2.813

**Published:** 2023-12-03

**Authors:** Somayeh Niakan, Hosseinali Mahgoli, Aylar Afshari, Seyed Ali Mosaddad, Aysooda Afshari

**Affiliations:** ^1^ Department of Prosthodontics, Dental Research Center, Faculty of Dentistry Tehran University of Medical Sciences Tehran Iran; ^2^ Student Research Committee, School of Dentistry Shiraz University of Medical Sciences Shiraz Iran; ^3^ Department of Conservative Dentistry and Bucofacial Prosthesis, Faculty of Odontology Complutense University of Madrid Madrid Spain; ^4^ Department of Prosthodontics, Faculty of Dentistry Yasuj University of Medical Sciences Yasuj Iran

**Keywords:** dental prosthesis, implant‐supported denture, overdenture, patient satisfaction

## Abstract

**Objectives:**

This study sought to determine the impact of age, gender, and use of maxillary implant‐supported overdenture (UISOD) versus conventional denture (CMD) opposing mandibular implant‐supported overdenture (LISOD) and the number of years the patient has used their prosthesis on the ratings of satisfaction. This study aimed to assess the long‐term influence of different factors on patients' satisfaction with LISODs needing a maxillary prosthesis, helping dentists choose a treatment pathway that leads to a higher satisfaction rate.

**Material and Methods:**

This cross‐sectional study included 84 participants. They were treated with LISOD opposing either CMD or UISOD from 2015 to 2020. They were all eligible to participate in the study. An oral health impact profile (OHIP‐14) questionnaire was given to each patient and filled out by them. OHIP‐14 scores were gathered and went under statistical analysis with PASS‐11 software to determine the relationship between patients' satisfaction with the scores.

**Results:**

Age and gender had no significant influence on how satisfied patients were. Patients with maxillary overdentures showed more satisfaction than the CMD group (*p* < .05). Moreover, patients' satisfaction decreased with increasing years of prostheses usage (*p* < .05).

**Conclusions:**

This study demonstrated that satisfaction is related to the type of maxillary prosthesis (conventional or implant‐supported) used opposing LISODs and the number of years the patient had used the prostheses.

## 1 INTRODUCTION

Although there has been a decline in edentulism, as the population of the elderly (over 65 years of age) is increasing worldwide, future predictions indicate that the number of patients in need of complete dentures will remain stable or even increase (Matthys, Vervaeke, Besseler, Doornewaard, et al., 2019). Conventional dentures were the sole treatment pathway for edentulism for over a century (Assunção et al., 2010).

Conventional dentures exhibit several clinical issues, such as pain, poor function, and a lack of retention and stability. They can also be related to reducing patients' confidence and comfort (Parel, 1986; van Waas, 1990). The research has examined several patient satisfaction factors related to aesthetics, speech, comfort, stability, and ease of chewing.

Additionally, the patient's gender, age, and previous prosthetic history should be considered when evaluating patient satisfaction (Siadat et al., 2008). Although the patient's ability to masticate and oral function is crucial in determining their level of satisfaction, these are not the only criteria. The level of patient satisfaction is a highly complex phenomenon that depends on many variables. Moreover, the patient's psychological status is essential to their overall satisfaction and adaptation to the complete denture. For example, the patient's emotional problems can impact the patient's rejection of the denture (Bergman & Carlsson, 1972; Boretti et al., 1995). Carlsson et al. (1967) showed that 10%–18% of patients remained unsatisfied with oral health after treatment with conventional dentures.

Conventional dentures can show variable rates of treatment success. It depends on the patient's adaptive capacity through a habituation process to face the limitations of complete dentures. Because of the inadequacy of conventional dentures, implant‐supported overdentures are used as an alternative. Implant‐supported overdentures improve denture retention, stability, patient satisfaction, and quality of life. After implant‐supported therapies have become a routine treatment modality, many problems of conventional treatments have been eliminated. The patient's satisfaction can determine the success of the treatment. Thus, evaluating the patient's satisfaction can confirm the value of therapy with implant‐supported overdenture (Assunção et al., 2010; Siadat et al., 2008). Moreover, differences between conventional maxillary dentures (CMDs) and maxillary implant‐supported overdentures (UISOD) opposing mandibular implant‐supported overdentures (LISOD) have not been studied in the literature. Thus, this paper aimed to evaluate patients' satisfaction with 1–5 years of CMD or UISOD uses opposing LISOD, including comfort, hygiene, oral function, esthetic, and general satisfaction considering various patient‐related factors such as age, gender, and past dental history.

## 2 MATERIALS AND METHODS

This study was a descriptive‐analytical cross‐sectional study. The recruiting process, exclusion criteria, and informed consent forms were all authorized by the university's Clinical Research Ethics Board under the ethical code IR.TUMS.DENTISTRY.REC.1400.002. Subjects were edentulous patients with CMD or UISOD opposing LISOD treated at the Department of Prosthetic of Dentistry at Tehran University of Medical Sciences between 2015 and 2020. The inclusion criteria were as below:

1. Healthy individuals (ASA I and II).
2. Mandibular condition: LISOD with two to four implants.
3. Maxillary condition: CMD or UISOD with four implants.
4. Type of attachments: Bar or stud attachments (locator, kerator, or ball).
5. Regular implant 10–13 mm long and 3.6–5 mm wide.
6. Implant systems: Dentium and Implantium (Dentium Co.), SIC (SIC Invent), DIO (DIO Corporation), or ITI (Institut Straumann AG).
7. Follow‐up durations of 1–5 years.

Exclusion criteria included compromised health status such as having any physical or systemic diseases, having parafunctional habits, having a history of head and neck radiotherapy, having a history of cognitive disorders that influence oral health indexes and oral health‐related quality of life (OHRQol), poor hygiene status, implants wider than 5 mm or narrower than 3.6 mm, and implants longer than 13 mm or shorter than 10 mm.

Archived records of patients treated at the Department of Prosthetic of Dentistry at Tehran University of Medical Sciences between 2015 and 2020 were retrieved and screened based on the inclusion and exclusion criteria. Then, patients who matched the eligibility criteria of this study were called, and the aim of this research was discussed with them. Willing patients were recalled for a follow‐up session. In that session, the age, gender, and previous dental history—including the duration of edentulism, the number of complete dentures worn before implant treatment, the number of implants used in implant‐supported overdenture treatment, and the number of adjustment appointments for included subjects—were first recorded with the aid of the patient's archived records.

In this study, the patient's satisfaction was evaluated by using the Persian translation of the “oral health impact profile” (OHIP‐14) questionnaire (Navabi et al., 2010). This questionnaire contains 14 questions and evaluates seven aspects of dentures and the patient's status: functional limitation, physical pain, psychological discomfort, physical disabilities, psychological disability, social disability, and handicap. Each 14 questions can be scored from 0 to 4 (0: *never*; 1: *seldom*; 2: *sometimes*; 3: *mostly*; 4: *always*). They can be calculated by adding up the values of all 14 items. The OHIP‐14 scores may range from 0 (*maximum satisfaction*) to 56 (*minimum satisfaction*). Higher OHIP‐14 values indicate a worse situation, whereas lower scores indicate a better OHRQol (Vinita Mary et al., 2017).

After signing consent forms, the questionnaires were given to the patients. The dentist asked questions individually and explained each question if necessary, and patients anonymously filled out the questionnaires. The examiner also conducted a comprehensive clinical examination to check the oral health of included subjects. Data from questionnaires were gathered and entered into an Excel workbook for statistical analysis.

Using statistical software of PASS‐11 with the usage of the McNemar option and consideration of *α* = .05, *β* = .2, and proportion discordant = 0.4 to find meaningful friction of 0.3, the minimum sample size was calculated as *n* = 36 (considering sample ratio 1:1), and *n*1 = 56, *n*2 = 28 (considering sample ratio 2:1; G1: CMD opposing LISOD and G2: UISOD opposing LISOD). OHIP‐14 value and the effects of age, sex, the type of maxillary denture, and duration of wearing prostheses on patient satisfaction were analyzed by Mann–Whitney, analysis of variance (ANOVA), and regression tests.

## 3 RESULTS

**Figure 1:**
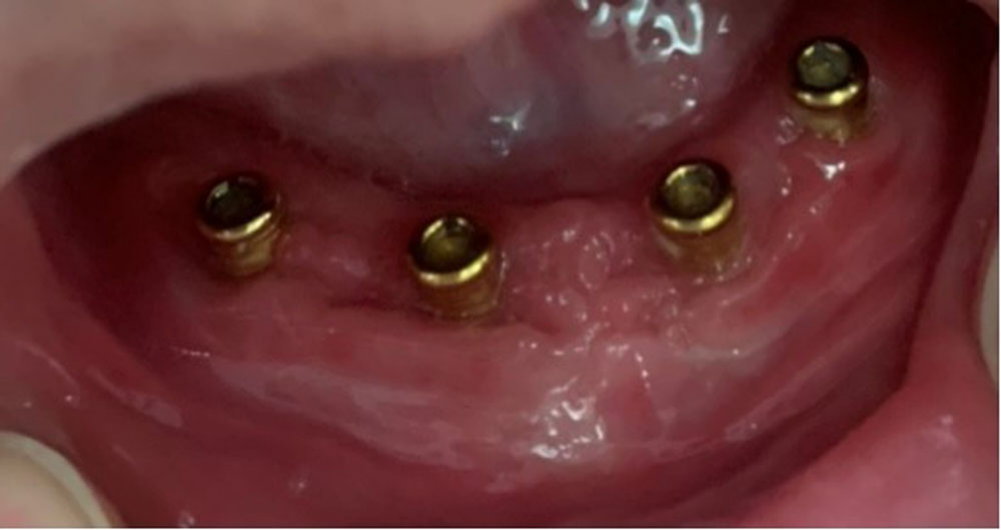
Stud attachments for supporting implant‐supported overdenture.

**Figure 2:**
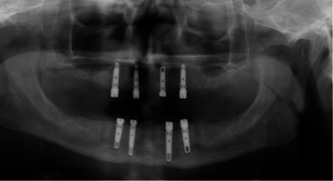
Maxillary and mandibular implant‐supported overdentures.

**Figure 3:**
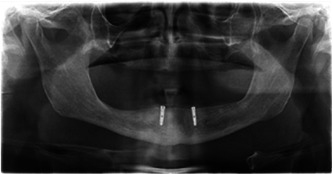
Maxillary conventional denture opposing mandibular implant‐supported overdenture.

Eighty‐four patients were analyzed in this study and were split into two groups. The first group included 56 patients having CMD opposing LISOD (Figures 1 and 2). The patient's ages ranged from 40 to 83 years old, with a mean of 62.63 years. Thirty‐two patients (57.1%) were male, and 24 (42.9%) were female in this study group. The patients used their prostheses for 1–5 years, with a mean value of 2.84 ± 1.218 years. The second group included 28 patients having UISOD opposing LISOD (Figure 3). The patient population ranged in age from 40 to 82, with a mean age of 63.29. Twelve (42.9%) female and 16 (57.1%) male patients made up group 2. The patients used their prostheses for 1–5 years, with a mean value of 2.75 ± 0.967.

The total number of dependent variables is mentioned in Table 1. The mean OHIP‐14 value for the first group was at intake 10.68 (SD 6.780, range: 4–32), and for the second group, 7.36 (SD 6.595, range: 3–26). Between the two study groups, there were statistically significant differences in patient satisfaction (*p* < .05), meaning that the type of maxillary prosthesis influenced patients' satisfaction and caused a meaningful difference in their comfort. With the regression and ANOVA analysis, the interaction of age, gender, maxillary prosthetics type, and years of prostheses usage with patient satisfaction were examined. It was observed that the age and gender of patients had no significant effect on patient satisfaction. Patients with maxillary overdentures significantly showed more satisfaction in comparison to the first group (*p* < .05); also, it was demonstrated that with increasing the years of prostheses usage, the satisfaction of patients had decreased (*p* < .05). Since all participants of this study were healthy individuals, no adverse health factors influencing the satisfaction ratings were observed.

**Table 1:** Coefficients: Total number of dependent variables.

Model		Unstandardized coefficients		Standardized coefficients	*t*	Significance
		*B*	SE	*β*		
1	(Constant)	−2.720	3.328		−0.817	0.416
	Age (years)	0.024	0.046	0.039	0.531	0.597
	Gender	0.232	1.093	0.017	0.212	0.832
	Prost. year	2.998	1.372	0.496	2.185	0.032
	Max. prost.	−6.127	3.039	−0.423	−2.016	0.047
	Interaction	1.149	1.029	0.326	1.116	0.268
2	(Constant)	−2.604	3.263		−0.798	0.427
	Age (years)	0.022	0.045	0.036	0.500	0.619
	Prost. year	3.063	1.329	0.507	2.305	0.024
	Max. prost.	−6.054	3.001	−0.418	−2.017	0.047
	Interaction	1.124	1.016	0.319	1.106	0.272
3	(Constant)	−1.162	1.517		−0.766	0.446
	Prost. year	3.054	1.323	0.505	2.309	0.024
	Max. prost	−6.020	2.986	−0.416	−2.016	0.047
	Interaction	1.117	1.012	0.317	1.104	0.273
4	(Constant)	−1.912	1.359		−1.407	0.163
	Prost. year	4.434	0.430	0.734	10.303	0.000
	Max. prost	−2.926	1.030	−0.202	−2.840	0.006

Abbreviations: max., maxillary; pros., prosthesis.

## 4 DISCUSSION

Conventional dentures have been the treatment for edentulous patients for more than 100 years. After implant‐supported overdentures have been introduced to the dental industry, they are slowly turning to an alternative to conventional dentures due to the increase in patients' comfort, satisfaction, oral function, and other physical and psychological improvements that they can cause. This cross‐sectional study compared patients' satisfaction with LISOD opposing two types of removable prostheses. The results of our study demonstrated that age and gender had no significant effect on patients' satisfaction. Also, with the increase of years of prosthesis usage, patient satisfaction decreases. Finally, patients with UISOD opposing LISOD showed more satisfaction and comfort than those with CMD opposing LISOD.

Although Müller & Hasse‐Sander (1993) explained that the performance of prostheses and oral tactile function decreases with age (therefore, decreasing satisfaction), age and functional features did not significantly correlate in the current investigation. Our result did not confirm with Hamlet et al.'s (1978) either, which indicated that the patient's age significantly impacted the patient's speech capacity and ability to adjust to a new prosthesis. Thomason et al.'s (2004) study demonstrated that older patients frequently preferred removable prostheses to fixed implant‐supported prostheses. Older patients with dexterity or vision loss usually find cleaning the abutments under a fixed restoration challenging. Siadat et al. (2008). assessed patient satisfaction with LISOD using a designed questionnaire.

Contrary to the findings of the present investigation, they concluded that satisfaction was connected with age, gender, and prior prosthetic history. This discrepancy might be brought about by the fact that in their study, there were significantly more male patients than female patients. Fernandez‐Estevan et al. (2017). showed that, in contrast to our study, the patient's age had an inverse relationship with impact on well‐being. Toia et al. (2019). studied the satisfaction rate of patients with implant‐supported overdentures. Their study came to a similar conclusion: patient satisfaction was not influenced by the patient's age or gender. The fact that there was no correlation between age and patient satisfaction in our study may be because most participants were older, with a mean age of more than 60. In studies with a higher age range, the relation between age and patients' satisfaction with their prostheses could be more significant.

Older patients over the age of 50 reported greater comfort when utilizing removable prostheses as opposed to fixed implant‐supported ones (Slade & Spencer, 1994). Like Thomason et al. (2004)., who studied the differences in patient satisfaction between a conventional denture and an overdenture supported by two implants with ball‐shaped retentive anchors, and Assunção et al. (2010), who conducted a literature review, our study suggests that usage of overdentures in comparison to conventional dentures (in the maxilla) causes more comfort in patients. This comfort can be due to better oral function, decreased pain, psychological discomfort, and social disability. Similar to this study, in a prospective study, Matthys et al. (2019). concluded from OHIP‐14 values that the usage of implant‐supported overdenture instead of conventional dentures improves patient satisfaction. Toia et al. (2019), similar to our study, showed that patients' satisfaction improves by using implant‐supported overdentures rather than conventional dentures. Al‐Zubeidi et al. (2012) assessed patient satisfaction with maxillary overdentures (supported by three implants) opposing mandibular overdentures (supported by two implants). They used visual analog scale questionnaires and the OHIP‐20. The satisfaction was evaluated at pretreatment (with conventional denture), with implant‐supported overdenture (baseline), and annually for 2 years after the treatment. Similar to this study, they found that patients with implant‐supported overdentures reported higher satisfaction levels than those with conventional dentures. Fernandez‐Estevan et al. (2017) evaluated the satisfaction of 80 patients who had worn mandibular overdentures supported by two implants for at least 1 year of function by using the OHIP‐20 and oral satisfaction scale (OSS). In contrast to the current investigation, they discovered that the kind of antagonist arch had no bearing on the degree of patient satisfaction. In conventional dentures, a condition similar to combination syndrome can occur. Combination syndrome mainly happens in patients with mandibular class I partial dentures opposing complete maxillary dentures. This condition may result in several complications, such as severe resorption of the premaxilla. It seems that this resorption causes the posterior part of the maxillary denture to be placed lower than expected, which can result in the enlargement of tuberosities. Tuberosity enlargement can be the reason for premature contact, decreased stability, and retention of the dentures, which may cause an increase in denture mobility, thus decreasing probably satisfaction of the patient. In contrast, it appears that if both maxilla and mandible prostheses are implant‐supported, the combination syndrome‐like condition does not occur, stability, retention, bilateral‐balance occlusion, the ease of mastication, and bite force increases, thus resulting in increased patient satisfaction.

Like this study, Fenlon and Sherrif (2004) and Berg (1988) showed that the patient's satisfaction regarding comfort with dentures decreases with increasing years of denture usage. Moreover, Fernandez‐Estevan et al. (2017) showed that the influence on well‐being is inversely correlated with prosthesis age. Contrarily, Matthys et al. (2019). concluded that satisfaction improves over time and is negatively related to maintenance costs. On the other hand, in Al‐Zubeidi et al.'s study (2012), in comparison to baseline values, no significant changes were seen in the first or second years. This difference between Al‐Zubeidi's study and the present study could be due to the difference between years of follow‐ups of each study. Al‐Zubeidi et al. had a 2‐year follow‐up, which, in contrast to our study, showed that this amount of time may not be enough to observe the true effects of years of prosthesis usage on patient satisfaction. As the years of prostheses usage increase, patients face more complications: the abrasion of denture teeth and denture mobility increases, posterior ridge resorption continues, and denture adaption decreases. Due to these factors, redenture may be necessary after several years of prosthesis usage. The presence of these complications and the cost of remaking the denture and follow‐ups may result in decreased satisfaction of the patients with increasing years of prostheses usage. In the treatment planning of patients, sociodental indicators are essential for evaluating the satisfaction of the oral condition. However, a simple measurement of satisfaction with prosthesis rehabilitation does not allow the complete assessment of dental care's effect on a person. Therefore, more clinical investigations are needed with a standardized analytical methodology to support or negate these results. The main limitation of this study was that because of the coronavirus disease 2019 pandemic, recalling the patients was challenging; due to this reason, it would be better to do a similar study with a higher number of participants.

## 5 CONCLUSION

In light of this study's shortcomings, it could be concluded that the age and gender of patients, although having influence, did not show any significant difference in the comfort and satisfaction of the patients. This study showed that UISOD opposing LISOD causes more satisfaction in patients of all ages and genders than CMD opposing LISOD. Furthermore, a decrease in satisfaction had been observed by increasing the age of denture usage of any kind in patients of all ages and genders.

## AUTHOR CONTRIBUTIONS

*Conceptualization and methodology*: Somayeh Niakan, Hosseinali Mahgoli, and Aysooda Afshari. *Data curation and formal analysis*: Aylar Afshari and Aysooda Afshari. *Investigation and resources*: Aylar Afshari and Aysooda Afshari. *Original draft preparation*: Aylar Afshari, Aysooda Afshari, and Seyed Ali Mosaddad. *Writing, reviewing, and editing*: Hosseinali Mahgoli and Seyed Ali Mosaddad. *Supervision and project administration*: Somayeh Niakan and Hosseinali Mahgoli.

## CONFLICT OF INTEREST STATEMENT

The authors declare no conflict of interest.

## ETHICS STATEMENT

The university's Clinical Research Ethics Board approved the research protocol with the ethical code of IR.TUMS.DENTISTRY.REC.1400.002, including recruitment procedures, exclusion/inclusion criteria, and informed consent. All individuals taking part in the study gave their informed consent.

## Data Availability

The data that support the findings of this study are available on request from the corresponding author.
